# PprA Protein Is Involved in Chromosome Segregation via Its Physical and Functional Interaction with DNA Gyrase in Irradiated *Deinococcus radiodurans* Bacteria

**DOI:** 10.1128/mSphere.00036-15

**Published:** 2016-01-13

**Authors:** Alice Devigne, Philippe Guérin, Johnny Lisboa, Sophie Quevillon-Cheruel, Jean Armengaud, Suzanne Sommer, Claire Bouthier de la Tour, Pascale Servant

**Affiliations:** a Institute for Integrative Biology of the Cell (I2BC), CEA, CNRS, Univ Paris-Sud, Université Paris-Saclay, Gif-sur-Yvette cedex, France; b CEA, DSV, IBiTec-S, SPI, Li2D, Laboratory Innovative Technologies for Detection and Diagnostics, Bagnols-sur-Cèze, France; University of Wyoming

**Keywords:** *Deinococcus radiodurans*, PprA, DNA gyrase, DNA decatenation

## Abstract

PprA, a radiation-induced *Deinococcus*-specific protein, was previously shown to be required for cell survival and accurate chromosome segregation after exposure to ionizing radiation. Here, we used an *in vivo* approach to determine, by shotgun proteomics, putative PprA partners coimmunoprecipitating with PprA when cells were exposed to gamma rays. Among them, we found the two subunits of DNA gyrase and, thus, chose to focus our work on characterizing the activities of the deinococcal DNA gyrase in the presence or absence of PprA. Loss of PprA rendered cells hypersensitive to novobiocin, an inhibitor of the B subunit of DNA gyrase. We showed that treatment of bacteria with novobiocin resulted in induction of the radiation desiccation response (RDR) regulon and in defects in chromosome segregation that were aggravated by the absence of PprA. *In vitro*, the deinococcal DNA gyrase, like other bacterial DNA gyrases, possesses DNA negative supercoiling and decatenation activities. These two activities are inhibited *in vitro* by novobiocin and nalidixic acid, whereas PprA specifically stimulates the decatenation activity of DNA gyrase. Together, these results suggest that PprA plays a major role in chromosome decatenation via its interaction with the deinococcal DNA gyrase when *D. radiodurans* cells are recovering from exposure to ionizing radiation.

**IMPORTANCE** *D. radiodurans* is one of the most radiation-resistant organisms known. This bacterium is able to cope with high levels of DNA lesions generated by exposure to extreme doses of ionizing radiation and to reconstruct a functional genome from hundreds of radiation-induced chromosomal fragments. Here, we identified partners of PprA, a radiation-induced *Deinococcus*-specific protein, previously shown to be required for radioresistance. Our study leads to three main findings: (i) PprA interacts with DNA gyrase after irradiation, (ii) treatment of cells with novobiocin results in defects in chromosome segregation that are aggravated by the absence of PprA, and (iii) PprA stimulates the decatenation activity of DNA gyrase. Our results extend the knowledge of how *D. radiodurans* cells survive exposure to extreme doses of gamma irradiation and point out the link between DNA repair, chromosome segregation, and DNA gyrase activities in the radioresistant *D. radiodurans* bacterium.

## Abstract (precis)

*D. radiodurans* is one of the most radiation-resistant organisms known. This bacterium is able to cope with high levels of DNA lesions generated by exposure to extreme doses of ionizing radiation and to reconstruct a functional genome from hundreds of radiation-induced chromosomal fragments. Here, we identified partners of PprA, a radiation-induced *Deinococcus*-specific protein, previously shown to be required for radioresistance. Our study leads to three main findings: (i) PprA interacts with DNA gyrase after irradiation, (ii) treatment of cells with novobiocin results in defects in chromosome segregation that are aggravated by the absence of PprA, and (iii) PprA stimulates the decatenation activity of DNA gyrase. Our results extend the knowledge of how *D. radiodurans* cells survive exposure to extreme doses of gamma irradiation and point out the link between DNA repair, chromosome segregation, and DNA gyrase activities in the radioresistant *D. radiodurans* bacterium.

## INTRODUCTION

The bacterium *Deinococcus radiodurans* possesses exceptional resistance to the lethal effects of DNA-damaging agents and is able to reconstruct a functional genome from a myriad of radiation-induced chromosomal fragments. This radioresistance is likely the result of a combination of different mechanisms, including protection of proteins against oxidation, efficient DNA double-strand break repair, and a compact nucleoid structure (for reviews, see references 1 to 6). Different DNA repair pathways have been proposed to be involved in the reconstitution of an intact genome in *D. radiodurans*, including extended synthesis-dependent strand annealing (ESDSA) (7), homologous recombination (HR) (8 – 10), single-strand annealing (SSA) (11 – 13), and nonhomologous end joining (14, 15).

The *pprA* gene (*DRA_0346*) is a *Deinococcus*-specific gene belonging to a radiation desiccation response (RDR) regulon comprising genes that are highly induced after DNA damage and contain a conserved motif (RDRM) upstream from their coding region (16, 17). Recently, it was shown that DdrO acts as a repressor of the RDR regulon and that IrrE, a metalloprotease, cleaves DdrO after irradiation, leading to transcriptional induction of various genes belonging to the RDR regulon (18 – 20). A *pprA* mutant exhibits high sensitivity to gamma radiation and DNA-damaging agents (14, 21, 22). *In vitro*, PprA preferentially binds double-stranded DNA (dsDNA) carrying strand breaks, inhibits *Escherichia coli* exonuclease III activity, and stimulates the DNA end-joining reaction catalyzed by ATP-dependent DNA ligases (14). It has also been shown that PprA polymerizes along supercoiled, nicked, circular, or linear double-stranded DNA (23). After irradiation, PprA is part of a multiprotein complex containing 24 proteins, including DNA ligases, DNA topoisomerase IB (Topo IB), SSB, and DNA polymerase I and exhibiting both DNA synthesis and DNA end-processing functions (24). We recently reported that repair of DNA double-strand breaks (DSB) in cells devoid of PprA and exposed to gamma radiation takes place efficiently, with a delay of approximately 1 h compared to the time for the wild type (21). All these results suggest that PprA might function as a pleiotropic protein involved in the repair of DNA DSB and other radiation-induced damage (6, 14). After irradiation, the PprA protein is recruited onto the nucleoid early and localizes later through the septum of dividing cells when DNA repair is completed (21). Untreated cells devoid of PprA display a wild-type morphology, but after gamma irradiation, the absence of PprA leads to severe defects in DNA segregation and cell division (21).

In bacteria, topoisomerases play a major role in chromosome segregation after completion of DNA replication. DNA topoisomerases are enzymes that resolve the topological transitions of DNA and are associated with replication, transcription, and recombination (for a review, see reference 25). They are divided into two types, depending on whether they operate by cleaving one strand and passing the other strand through the break (type I) or by cleaving both strands and passing a DNA duplex through the DNA double-strand break (type II). Most bacteria possess at least three DNA topoisomerases, one type I enzyme, DNA topoisomerase I (Topo I), encoded by the *topA* gene, and two type II enzymes, DNA gyrase and DNA topoisomerase IV (Topo IV), which are heterotetramers with two different subunits, encoded by the *gyrA* and the *gyrB* genes and by the *parC* and *parE* genes, respectively. DNA topoisomerase I relaxes DNA, while DNA gyrase introduces negative DNA supercoils. These opposing activities allow the maintenance of DNA superhelicity in the cells. DNA topoisomerase I and DNA gyrase also act in concert to resolve topological constraints during replication and transcription. Because of these important physiological roles, DNA topoisomerase I and DNA gyrase are essential proteins for the viability of bacterial cells (26 – 29). Topo IV is involved in decatenation of intertwined DNA intermediates generated during DNA replication and DNA recombination (30, 31) and plays a major role in decatenation of daughter chromosomes before cell division (for reviews, see references 25, 32, and 33). Some bacteria, such as *Escherichia coli*, possess, in addition to these three topoisomerases, a DNA topoisomerase III (Topo III), which is another type IA topoisomerase encoded by the *topB* gene that might play a role in the unlinking of the DNA strands at the end of replication (34).

*D. radiodurans* has an atypical content of DNA topoisomerases: it possesses a DNA topoisomerase I, encoded by the *DR_1374* (*topA*) gene, and a DNA gyrase, encoded by the *DR_1913* (*gyrA*) and *DR_0906* (*gyrB*) genes. DNA gyrase is the only type II DNA topoisomerase in this bacterium. Indeed, the sequence of the *D. radiodurans* genome does not reveal any homolog of the *parC* or *parE* genes that encode the two subunits of Topo IV in *E. coli*. Like some other bacteria, *D. radiodurans* also possesses another topoisomerase, encoded by the *DR_0690* gene (*topIB* gene) and belonging to the type IB family, which includes eukaryotic nuclear topoisomerases and topoisomerases IB of poxviruses (35). *D. radiodurans* DNA gyrase was shown to be one of the main proteins involved in the organization of the *Deinococcus* nucleoids (36), but its biochemical activities have not been extensively characterized. Recently, Kota et al. (37) showed, using a bacterial two-hybrid system, that PprA interacts with DNA Topo IB and DNA gyrase from *D. radiodurans* and enhances the relaxation activity of Topo IB. They also showed that Δ*pprA* mutant bacteria are sensitive to nalidixic acid, an inhibitor of type II bacterial DNA topoisomerases (37).

Here, in order to decipher the role of PprA in chromosome segregation, we used an *in vivo* approach to determine by shotgun proteomics putative PprA partners interacting and coimmunoprecipitating with PprA after exposure to gamma radiation. We found among them the two subunits of DNA gyrase, and we decided to focus our work on characterizing the activities of the deinococcal DNA gyrase in the presence or absence of PprA. We also showed that treatment of bacteria with novobiocin, an inhibitor of DNA gyrase, resulted in defects in chromosome segregation that were aggravated by the absence of PprA. Our results suggest that PprA plays a major role in chromosome decatenation via its interaction with the deinococcal DNA gyrase when cells are recovering from exposure to ionizing radiation.

## RESULTS

### Shotgun MS highlights gyrase subunit A as a partner of PprA protein in gamma-irradiated *D. radiodurans* cells.

We have recently shown that PprA protein may play an important role in *D. radiodurans* by regulating chromosome segregation and the restart of cell division after the completion of DNA repair (21). Here, we used proteomic assays to identify possible PprA partners. The interacting proteins were trapped by coimmunoprecipitation with PprA. Immunoprecipitation was carried out using strain GY14615, which expresses a functional PprA::HA fusion protein from the native *pprA* promoter (21). Prior to immunoprecipitation, the cells were exposed to 3.8 kGy gamma radiation and allowed to recover in fresh medium for a period of 110 min. This procedure allowed an optimal induction of the PprA::HA protein, which is poorly expressed in nonirradiated cells (21). Furthermore, some partners could interact with PprA only after DNA damage.

Three independent biological replicates were performed using irradiated or nonirradiated cells expressing a PprA::HA fusion protein, and two independent biological replicates were performed using irradiated or nonirradiated Δ*pprA* cells as a negative control. Immunoprecipitated complexes obtained using anti-HA monoclonal antibodies from test and control samples were subjected to shotgun tandem mass spectrometry (MS-MS) analysis with a high-resolution orbitrap mass analyzer in order to identify and quantify the enriched proteins.

The entire set of proteins detected in the immunoprecipitated samples after irradiation is given in Table S1 in the supplemental material. It comprises 102 polypeptides uncovered through the assignment of 416 unique peptide sequences, 57 being certified by tandem mass spectrometry with at least two peptides detected. We first compared, by label-free proteomics, the protein coprecipitated with PprA::HA after gamma irradiation and the nonirradiated control, in which PprA::HA protein is expressed at its basal level (comparison 1). As expected, the PprA::HA protein was identified through an average of only 11 spectral counts when the cells were nonirradiated, while an average of 93 PprA::HA spectral counts was found for irradiated cells. Comparison 1 allowed the identification of proteins that accumulate in response to the irradiation. To identify proteins interacting specifically with PprA, we compared the proteins that coprecipitated with PprA::HA protein after exposure to 3.8 kGy gamma radiation to the proteins from Δ*pprA* bacteria exposed to the same dose of irradiation (comparison 2). Proteins detected in both comparisons were considered putative interacting partners of the PprA protein. Table 1 shows the eight most relevant proteins, which had changes of twofold or more in both comparisons, pointing out that they interacted with PprA::HA. Among them, elongation factor Tu and ribosomal protein S1 are implicated in translation, Hpi is involved in the maintenance and integrity of the S layer, and RNA helicase participates in nearly all aspects of RNA metabolism. We also found the substrate-binding subunit of a peptide ABC transporter, the chaperon protein GroEL, and the two subunits of DNA gyrase. We therefore focused our attention on DNA gyrase as a PprA interacting partner.

**TABLE 1:** Proteins that coprecipitated with PprA::HA protein after gamma irradiation

Gene	Function	Change in protein expression for^a^:			
		Comparison 1 (*pprA*::HA strain at 3.8 kG versus 0 kGy)		Comparison 2 (*pprA*::HA strain versus Δ*pprA* strain at 3.8 kGy)	
		Fold change	*P* value	Fold change	*P* value
*DR_0309*	Elongation factor Tu	7.2	1.95 × 10^−2^	7.2	3.96 × 10^−2^
*DR_2508*	Hexagonally packed intermediate layer surface protein	7.0	4.97 × 10^−4^	2.1	8.69 × 10^−2^
*DR_1913*	DNA gyrase subunit A	4.8	6.90 × 10^−2^	4.8	1.12 × 10^−1^
*DR_1983*	30S ribosomal protein S1	2.9	6.88 × 10^−2^	2.5	2.40 × 10^−1^
*DR_1624*	RNA helicase	2.6	2.18 × 10^−1^	2.6	2.90 × 10^−1^
*DR_1955*	Peptide ABC transporter substrate-binding protein	2.4	2.49 × 10^−2^	2.4	3.25 × 10^−1^
*DR_0607*	Chaperonin groEL	2.4	2.49 × 10^−2^	2.4	3.25 × 10^−1^
*DR_0906*	DNA gyrase subunit B	2.0	3.27 × 10^−2^	2.0	4.10 × 10^−1^

^a^ *D. radiodurans* Δ*pprA* (GY12251) and *pprA*::HA (GY14615) bacteria were either exposed or not to gamma irradiation (3.8 kGy) and allowed to recover in fresh medium for 110 min prior to performing a coimmunoprecipitation with anti-HA antibodies. Immunoprecipitated materials were analyzed by mass spectrometry (see Table S1 in the supplemental material). The comparison between PprA-HA samples exposed to gamma irradiation (3.8 kGy, five replicates) and nonirradiated samples (0 kGy, four replicates) was performed with the Tfold module. The comparison between gamma-irradiated samples (3.8 kGy) of the strain expressing PprA::HA (five replicates) and the *pprA* deletion mutant (two replicates) was performed with the ACfold module using the AC test described in Audic and Claverie (66). The eight proteins detected by shotgun proteomics with a >twofold increase after irradiation (comparison 1) and with a >twofold decrease after deletion of *pprA* (comparison 2) are listed.

10.1128/mSphere.00036-15.7 Table S1  List of identified proteins ranked by class and fold change in expression after irradiation. Download Table S1, XLSX file, 0.1 MB. Copyright © 2016 Devigne et al. 2016 Devigne et al. This content is distributed under the terms of the Creative Commons Attribution 4.0 International license.

In order to confirm the physical interaction between PprA protein and the GyrA subunit highlighted by mass spectrometry, we performed a cross-immunoprecipitation experiment using a strain expressing PprA and GyrA tagged with different epitopes (hemagglutinin [HA] and SPA [sequential peptide affinity], respectively). Both tagged proteins were functional, as shown by the viability and the wild-type radioresistance of the engineered strain (see Fig. S1A in the supplemental material). Immunoprecipitation was carried out on cell extracts from nonirradiated cells or from cells exposed to 3.8 kGy gamma radiation and allowed to recover for 110 min. Two different immunoprecipitations were performed, as follows: (i) the cell extracts were incubated with anti-HA antibodies and the bound proteins were separated by SDS-PAGE and immunoblotted with anti-FLAG antibodies to reveal the GyrA::SPA protein, and (ii) the cell extracts were incubated with anti-FLAG antibodies and the bound proteins were separated by SDS-PAGE and immunoblotted with anti-HA antibodies to reveal the PprA::HA protein.

10.1128/mSphere.00036-15.1 Figure S1  Survival of wild-type, *ΔpprA*, GY14697 (*pprA*::HA::*kan gyrA*::SPA::*cat*), and *ΔtopIB* strains after gamma ray irradiation. (A) Closed squares, wild type; open circles, GY14697; open triangles, *ΔpprA* strain. (B) Closed squares, wild type; open circles, *ΔtopIB* strain. Download Figure S1, EPS file, 0.7 MB. Copyright © 2016 Devigne et al. 2016 Devigne et al. This content is distributed under the terms of the Creative Commons Attribution 4.0 International license.

**FIG 1:**
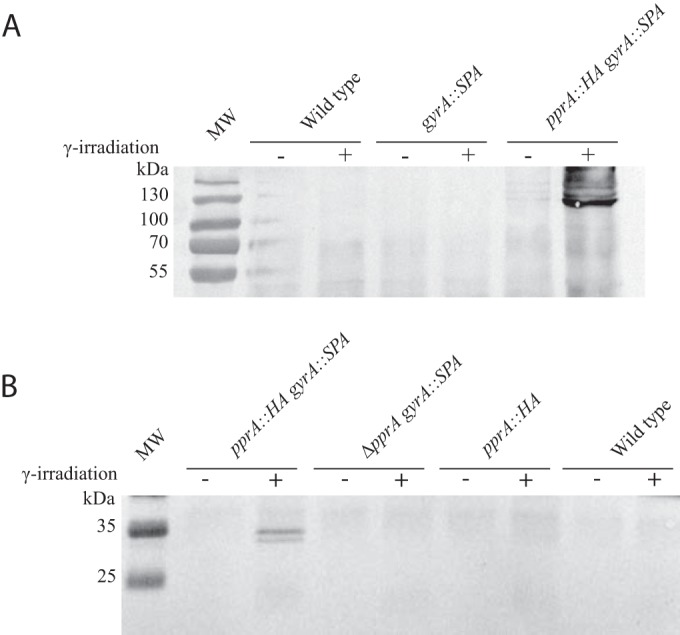
The GyrA subunit of DNA gyrase interacts with the PprA protein after irradiation. GY9613 (wild-type), GY13338 (*gyrA*::SPA), GY14641 (Δ*pprA gyrA*::SPA), GY14615 (*pprA*::HA), and GY14697 (*pprA*::HA *gyrA*::SPA) cells were exposed to 3.8 kGy gamma radiation and grown for 110 min. Cells were fixed and immunoprecipitation performed. (A) Anti-HA antibodies were used to capture HA-tagged PprA protein by immunoprecipitation. Eluted samples were separated on 10% SDS-PAGE gels, and primary anti-FLAG antibodies were used in Western blot analyses to reveal SPA-tagged GyrA proteins. (B) Anti-FLAG antibodies were used to capture SPA-tagged GyrA proteins by immunoprecipitation, eluted samples were separated on 12% SDS–PAGE, and primary anti-HA antibodies were used in Western blots to reveal HA-tagged PprA proteins.

To check the specificity of our system, control experiments were performed with strains that did not express the PprA::HA protein (*gyrA*::SPA strain) (Fig. 1A) or the GyrA::SPA protein (*pprA*::HA strain) (Fig. 1B). After coimmunoprecipitation of proteins from these irradiated or nonirradiated *gyrA*::SPA and *pprA*::HA control strains, no signal was detected with anti-FLAG antibodies (Fig. 1A) or anti-HA antibodies (Fig. 1B), respectively. Our results obtained with the *pprA*::HA *gyrA*::SPA strain confirm a physical interaction between PprA and GyrA proteins *in vivo*. Indeed, the GyrA protein was specifically trapped by immunoprecipitation of PprA::HA (Fig. 1A), and conversely, the PprA protein was specifically trapped by immunoprecipitation of GyrA::SPA (Fig. 1B). In the results shown in Fig. 1A, we also observed additional bands that may correspond to GyrA::SPA cross-linked with other proteins. The interaction between the two proteins was detected only after exposure of the cells to gamma irradiation, suggesting that PprA is not expressed at a sufficient level in nonirradiated cells to allow the detection of this interaction or that the interaction between the two proteins only takes place during recovery from DNA damage.

### Loss of PprA renders *D. radiodurans* cells hypersensitive to DNA gyrase inhibitors.

**FIG 2:**
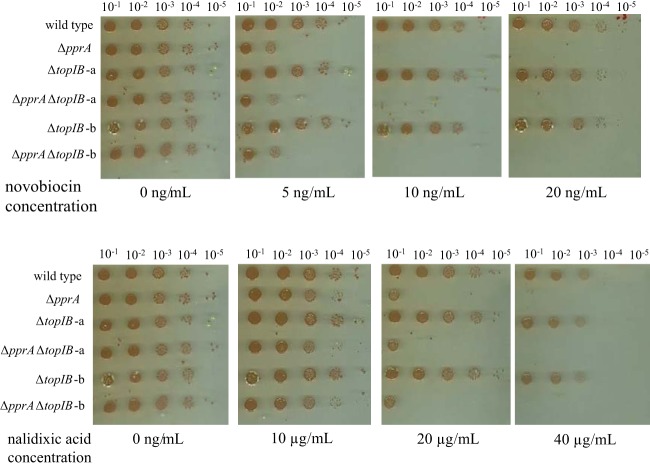
Sensitivity of Δ*pprA* mutant to gyrase inhibitors. Serial dilutions of cultures of GY9613 (wild-type), GY14661 (Δ*pprA*), GY15987 (Δ*topIB*) (2 clones, a and b), and GY15985 (Δ*pprA* Δ*topIB*) (2 clones, a and b) strains were spotted on plates in the presence or absence of novobiocin or nalidixic acid at the indicated concentrations, and plates were incubated at 30°C for 5 days.

The above-described results suggest that PprA might act with DNA gyrase after irradiation. Since the *gyrA* and *gyrB* genes are essential (29, 36), we decided to use drugs that inhibit DNA gyrase activity to examine the effects of a *pprA* deletion in a gyrase-defective context. As previously shown (37), Δ*pprA* cells are hypersensitive to nalidixic acid, which targets the GyrA subunit of DNA gyrase (Fig. 2). Here, we showed that Δ*pprA* cells are also hypersensitive to novobiocin, a drug that targets the GyrB subunit of DNA gyrase (Fig. 2). Kota et al. showed that PprA also interacts with Topo IB and enhances the relaxation activity of this enzyme *in vitro* (37). In our study, we did not find Topo IB as an interacting partner of PprA. Moreover, Δ*topIB* and Δ*pprA* Δ*topIB* bacteria showed the same sensitivity to nalidixic acid or novobiocin as wild-type and Δ*pprA* bacteria, respectively (Fig. 2). The Δ*topIB* bacteria also showed a wild-type level of radioresistance (see Fig. S1B in the supplemental material). Our *in vivo* results suggest that Topo IB in *D. radiodurans* does not play a crucial role in DNA repair*.*

### Defects in segregation of cells exposed to novobiocin are increased in Δ*pprA* mutants.

**FIG 3:**
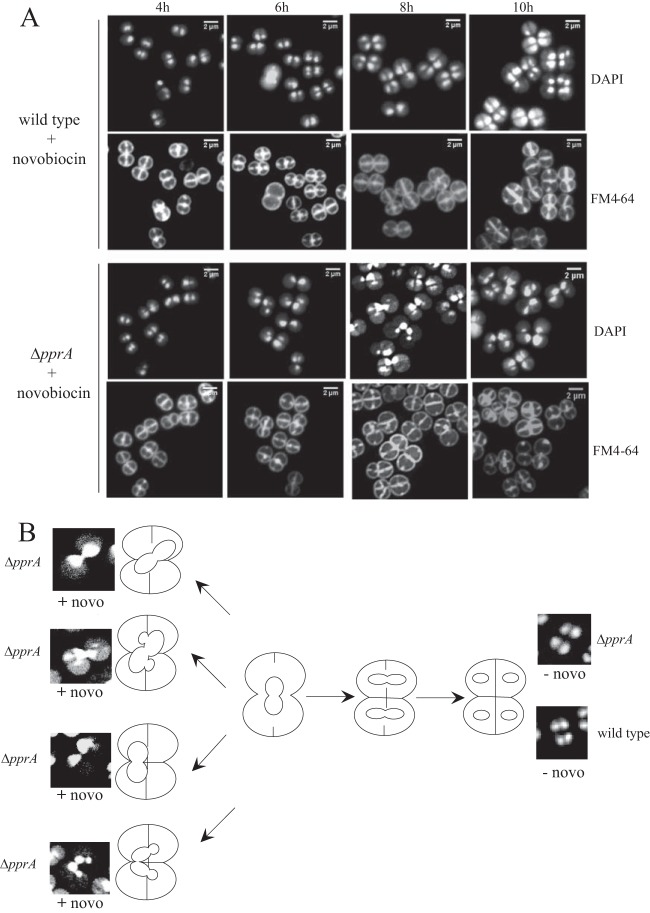
Morphologies of wild-type and Δ*pprA* mutant cells grown in the presence of novobiocin. (A) Cells were grown in the presence of 40 ng/ml novobiocin. Samples were taken at the indicated times after the addition of novobiocin and examined by fluorescence microscopy. DNA was stained with DAPI, and membranes were stained with FM 4-64. (B) Various abnormal forms of Δ*pprA* nucleoids observed after 8 h of growth in the presence of novobiocin. The wild-type and Δ*pprA* controls represent the cells grown without novobiocin. A schematic representation of the *D. radiodurans* dividing cells is also shown.

An analysis of cell morphology during the recovery of *D. radiodurans* cells from gamma irradiation showed that a PprA deficiency results in defective chromosome segregation and aberrant cell division (21). In *E. coli*, the Topo IV enzyme plays a key role in chromosome segregation by resolving catenated chromosomes generated during replication (30). *D. radiodurans* naturally lacks Topo IV, and thus, DNA gyrase is the only type II topoisomerase present in the cells and is probably involved in the chromosome decatenation process. We expected that inhibition of DNA gyrase by drugs would result in defective chromosome partitioning. This defect could be exacerbated by a PprA deficiency, accounting for the hypersensitivity of Δ*pprA* bacteria to nalidixic acid and novobiocin. Therefore, we analyzed the cell morphologies after treatment with novobiocin. For this purpose, samples of wild-type and Δ*pprA* cultures were taken at different times after the addition of novobiocin and analyzed by fluorescence microscopy (Fig. 3).

No aberrant cell morphologies were observed in the absence of novobiocin for either Δ*pprA* or wild-type cells (see Fig. S2 in the supplemental material). In contrast, treatment of the wild-type strain with novobiocin resulted in morphological abnormalities, including 9.6% of cells being anucleated (39/404) or cells having defects in segregation of the nucleoids (33/404 cells with unequal distribution of DNA between daughter cells or DNA blocked across the septum). The absence of the PprA protein aggravated the morphological defects visualized after novobiocin treatment (Fig. 3), with 30.8% of cells being anucleated (136/441) or cells having defects in segregation of nucleoids (22.2%, 98/441 cells) or defects in cell division, with cells being incompletely separated. Some examples of segregation and division defects are schematized in Fig. 3B. Large-field images confirmed that these abnormalities were present in >80% of the Δ*pprA* cells treated with novobiocin (see Fig. S2).

10.1128/mSphere.00036-15.2 Figure S2  Large-field images of wild-type and Δ*pprA* cells grown for 8 h with (upper panels) or without (lower panels) novobiocin. Nucleoids were stained with DAPI. Download Figure S2, EPS file, 5.1 MB. Copyright © 2016 Devigne et al. 2016 Devigne et al. This content is distributed under the terms of the Creative Commons Attribution 4.0 International license.

### Novobiocin treatment induces the RDR regulon.

As novobiocin treatment induces the SOS system in *Bacillus subtilis* (38), we tested the effect of novobiocin on the radiation desiccation response (RDR), the major response to DNA damage generated by ionizing radiation in *D. radiodurans* (16, 17). The induction of the RDR regulon results from proteolytic degradation of the DdrO repressor by an activated form of the metalloprotease IrrE (18, 19).

**FIG 4:**
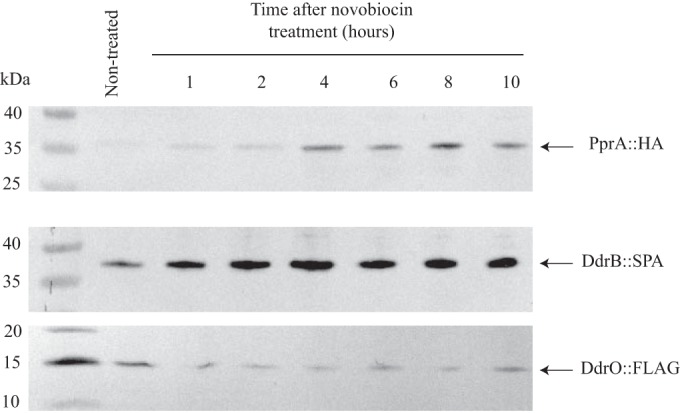
PprA and DdrB are induced, whereas the DdrO concentration decreases after novobiocin treatment. *D. radiodurans* GY14615 (*pprA*::HA), GY12830 (*ddrB*::SPA), and GY16173 (*ddrO*::FLAG) cells were incubated in the presence or absence of novobiocin for the indicated times. Cell extracts were subjected to SDS-PAGE and analyzed by Western blotting with anti-HA antibodies for PprA::HA detection and anti-FLAG antibodies for DdrB::SPA and DdrO::FLAG detection.

To test whether novobiocin treatment induced the RDR regulon, we examined, in the presence or absence of novobiocin, the levels of three proteins encoded by genes belonging to the regulon, namely, PprA and DdrB, whose expression is highly induced during the radiation desiccation response (RDR), and DdrO, whose level was expected to decrease during the course of the novobiocin treatment. The PprA::HA, DdrB::SPA, and DdrO::FLAG proteins were visualized by Western blot analysis using anti-HA or anti-FLAG antibodies. As can be seen by the results in Fig. 4, a faint band corresponding to the PprA::HA protein was detected in nontreated cells, but the signal increased markedly during the treatment with novobiocin. The results also clearly indicate that the DdrB::SPA protein was induced at early times after the addition of novobiocin (Fig. 4). In contrast, we observed a quick decrease in the level of the DdrO repressor in novobiocin-treated cells (Fig. 4). From these results, we conclude that novobiocin induces the RDR regulon in *D. radiodurans*.

### PprA stimulates decatenation activity of *D. radiodurans* DNA gyrase.

Our *in vivo* data suggest that PprA might interact with GyrA and modulate the DNA gyrase activities in cells recovering from DNA damage. To test this possibility, we sought to determine the effects of purified PprA protein on the *in vitro* activities of the deinococcal DNA gyrase. The DNA gyrase is the sole type II topoisomerase encoded by the *D. radiodurans* genome and is expected to possess both relaxing/supercoiling and catenation/decatenation activities that are also ensured by other bacterial DNA gyrases (for a review, see reference 39).

We first purified the two subunits of the deinococcal DNA gyrase. The GyrA (as a C-terminally 6His-tagged protein) and GyrB (as an N-terminally 6His-tagged protein) subunits of DNA gyrase were overproduced in *E. coli*, purified (see Fig. S3 in the supplemental material), and mixed in equimolar amounts to reconstitute the DNA gyrase holoenzyme.

10.1128/mSphere.00036-15.3 Figure S3  Purification of GyrA, GyrB, and PprA proteins. SDS-PAGE analysis of purified recombinant GyrA, GyrB, and PprA proteins. Amount of approximately 1 µg of GyrA and GyrB proteins and 10 µg of PprA protein were loaded on an SDS–10% polyacrylamide gel. Proteins were revealed by staining with Coomassie blue. The sizes of protein markers (M) are indicated to the left. Download Figure S3, EPS file, 1.4 MB. Copyright © 2016 Devigne et al. 2016 Devigne et al. This content is distributed under the terms of the Creative Commons Attribution 4.0 International license.

**FIG 5:**
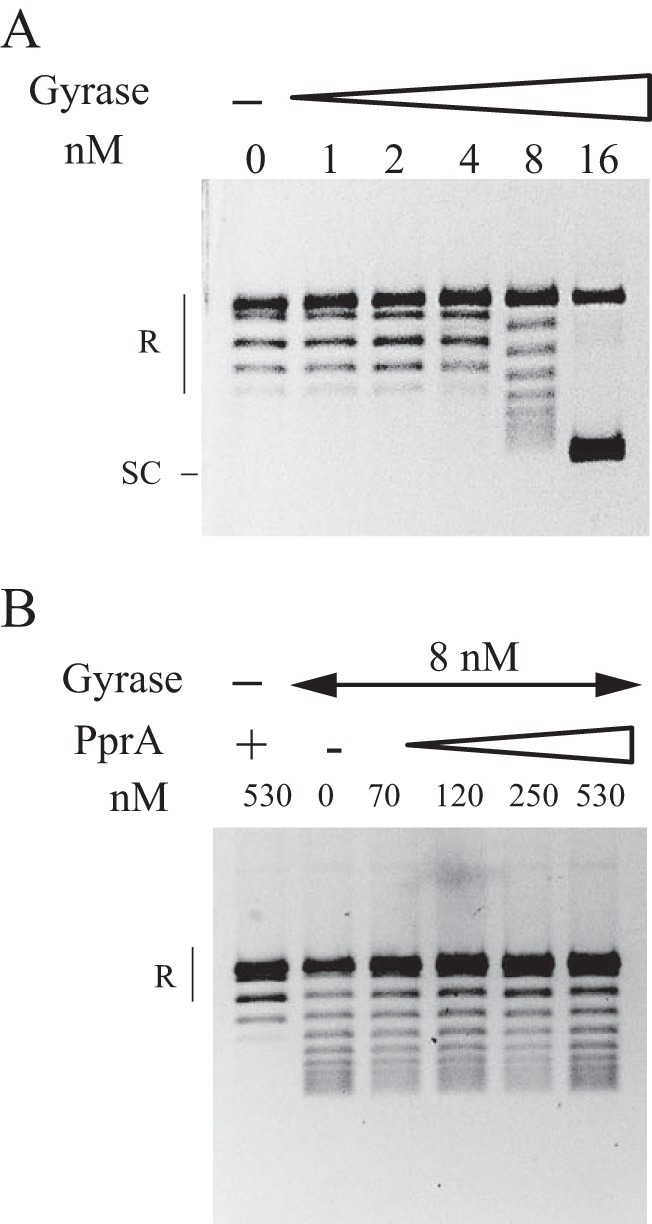
Assay for the DNA negative supercoiling activity of the *D. radiodurans* DNA gyrase. DNA gyrase was reconstituted by mixing the GyrA and GyrB subunits in equimolar amounts. (A) Relaxed pHOT plasmid DNA was incubated with the indicated increasing concentrations of DNA gyrase. SC, supercoiled plasmid DNA; R, relaxed plasmid DNA. (B) Effect of PprA on the DNA negative supercoiling activity of DNA gyrase. Relaxed pHOT DNA was incubated with DNA gyrase (8 nM) in the presence of the indicated increasing concentrations of PprA.

**FIG 6:**
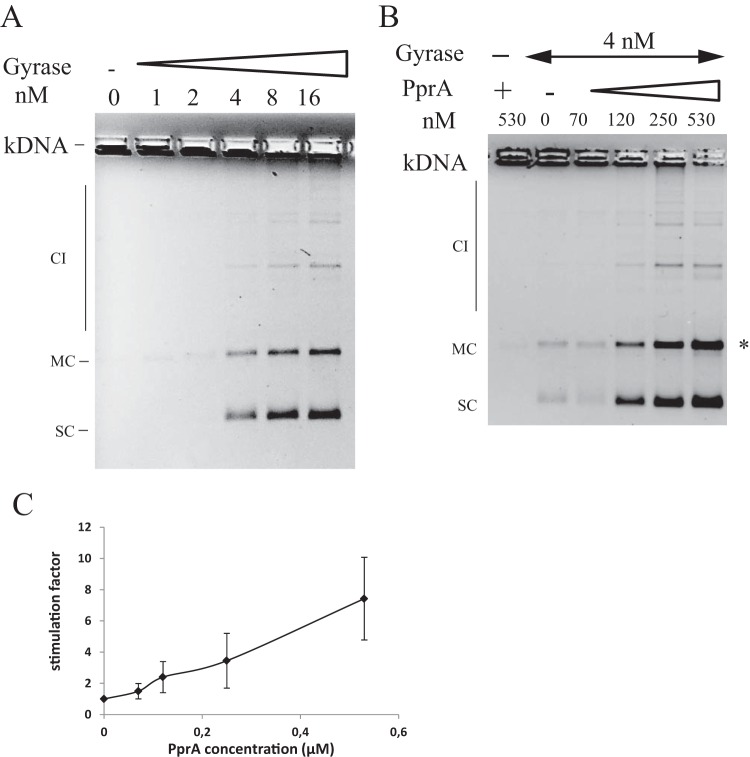
PprA stimulates decatenation activity of *D. radiodurans* DNA gyrase. (A) kDNA was incubated with the indicated increasing concentrations of DNA gyrase. kDNA was retained in the wells of the agarose gel. (B) Effect of PprA on the decatenation activity of DNA gyrase. DNA gyrase (4 nM) was incubated with kDNA in the presence of the indicated increasing concentrations of PprA. (C) Stimulation of DNA decatenation by PprA. The ratio of the minicircles released in the presence of PprA over the minicircles released in the absence of PprA was determined at each PprA concentration, and the average results from four independent experiments were plotted. SC, supercoiled minicircles; MC, minicircles; CI, catenated intermediates.

The DNA negative supercoiling activity of the DNA gyrase was measured using relaxed pHOT DNA as the substrate. The combination of GyrA and GyrB subunits led to the formation of intermediate topoisomers and supercoiled DNA when the concentration of DNA gyrase increased (Fig. 5A). The decatenation activity was tested by using the catenated kinetoplast DNA (kDNA; see Materials and Methods) as a substrate that would allow the unlinking of DNA minicircles to be monitored. We observed that DNA gyrase of *D. radiodurans* was able to decatenate the kDNA (Fig. 6A). A supercoiled form of DNA minicircles was also produced, due to the supercoiling activity of DNA gyrase (Fig. 6A). Neither the GyrA nor the GyrB subunit alone exhibited DNA supercoiling or decatenation activities, indicating that the activities observed in our assays were not due to contamination by DNA gyrase or Topo IV from the *E. coli* host (see Fig. S4 in the supplemental material).

10.1128/mSphere.00036-15.4 Figure S4  Absence of supercoiling and decatenation activities of the GyrA and GyrB subunits. (A) In the assay for DNA negative supercoiling activity, proteins were incubated with relaxed pHOT DNA plasmid as described in Materials and Methods. Lane A, GyrA subunit (16 nM); lane B, GyrB subunit (16 nM); lane AB, reconstituted DNA gyrase (8 nM); lane T, relaxed pHOT DNA control; R, relaxed plasmid DNA; SC, supercoiled plasmid DNA. (B) In the assay for decatenation activity, proteins were incubated with kDNA as described in Materials and Methods. Lane A, GyrA subunit (16 nM); lane B, GyrB subunit (16 nM); lane AB, reconstituted DNA gyrase (8 nM); lane T, kDNA control; MC, minicircles; CI, catenated intermediates; SC, supercoiled minicircles. Download Figure S4, EPS file, 2.1 MB. Copyright © 2016 Devigne et al. 2016 Devigne et al. This content is distributed under the terms of the Creative Commons Attribution 4.0 International license.

Furthermore, we tested the supercoiling and decatenation activities of DNA gyrase in the presence of increasing concentrations of novobiocin or nalidixic acid, and we showed that the two activities were inhibited by the drugs *in vitro* (see Fig. S5).

10.1128/mSphere.00036-15.5 Figure S5  Effects of novobiocin and nalidixic acid on *D. radiodurans* gyrase activities. Assays of supercoiling activity (upper panels) and decatenation activity (lower panels) were carried out in the presence of 16 nM DNA gyrase and increasing concentrations of novobiocin (left panels, lanes 2 to 6, 0, 0.16, 0.32, 0.64, and 1.2 µg/ml, respectively) or nalidixic acid (right panels, lanes 2 to 6, 0, 0.16, 0.32, 0.64, and 1.2 mg/ml, respectively). Lanes 1 represent the relaxed DNA control and the kDNA control incubated in the absence of DNA gyrase with the highest concentration of drugs, in the upper and lower panels, respectively. Download Figure S5, EPS file, 4.1 MB. Copyright © 2016 Devigne et al. 2016 Devigne et al. This content is distributed under the terms of the Creative Commons Attribution 4.0 International license.

To test the effects of PprA on the DNA gyrase activities, we purified a PprA::6His protein (see Fig. S3 in the supplemental material). In order to verify that the recombinant His-tagged PprA protein was functional, we constructed a *D. radiodurans* strain expressing the PprA::6His protein by allelic replacement of the wild-type *pprA* gene with its tagged counterpart. We showed that these bacteria were as resistant to nalidixic acid as were the wild-type bacteria (see Fig. S6), demonstrating the functionality of the tagged PprA protein. Increasing amounts of the purified PprA::6His protein were included in the supercoiling or decatenation reactions. As can be seen by the results in Fig. 5B, PprA had no effect on the negative supercoiling activity of DNA gyrase. In contrast, it stimulated the decatenase activity of DNA gyrase. Indeed, PprA increased the amount of the decatenated minicircle products in the presence of DNA gyrase in a dose-dependent manner (about a sevenfold increase at the highest PprA concentration) (Fig. 6B and C).

10.1128/mSphere.00036-15.6 Figure S6  The tagged PprA::6His protein is functional *in vivo*. Serial dilutions of bacterial cultures of the wild-type (GY9613), Δ*pprA* (GY14661), and *pprA*::6His (GY16179) strains were spotted on plates in the presence of nalidixic acid at 20 µg/ml, and the plates were incubated at 30°C for 5 days. Download Figure S6, EPS file, 1.2 MB. Copyright © 2016 Devigne et al. 2016 Devigne et al. This content is distributed under the terms of the Creative Commons Attribution 4.0 International license.

## DISCUSSION

The *pprA* gene is a *Deinococcus*-specific gene that is highly induced after exposure to ionizing radiation or desiccation, and its deletion renders *D. radiodurans* mutant cells highly sensitive to gamma irradiation. Remarkably, the major phenotypes observed in cells devoid of PprA after exposure to gamma radiation are defects in DNA segregation and cell division after completion of DNA repair (21). In wild-type cells, PprA is recruited onto the nucleoid early after irradiation and localizes later through the septum of dividing cells when DNA repair is completed (21). Here, we determined, by coimmunoprecipitation and shotgun proteomics, the direct or indirect interactants of PprA in cells exposed to 3.8 kGy gamma irradiation *in vivo*. Among the PprA interactants, we found the GyrA and the GyrB proteins. These results are in agreement with the direct interaction of PprA with the GyrA subunit of DNA gyrase that was observed previously using an *E. coli* bacterial two-hybrid system (37).

After gamma irradiation, PprA has been found in a multiprotein complex containing a few known DNA repair proteins (DNA ligases, SSB, nucleases, and DNA polymerase I), a molecular chaperone (DnaK), a DNA topoisomerase (Topo IB) and a large number of uncharacterized proteins (24). These proteins were not found in our study, but the conditions used by Kota et al. (37) (irradiation doses and postirradiation incubation time) were not explicitly defined, which renders a comparison of results somewhat difficult. Intriguingly, they did not find DNA gyrase in their complex. More recently, it was shown, using a bacterial two-hybrid system, that PprA interacts with Topo IB and enhances the Topo IB-mediated relaxation activity of negative supercoiled DNA (37). The demonstration of a structural and mechanistic relationship between topoisomerase IB and site-specific recombinases suggests a possible role of Topo IB in recombination in *D. radiodurans* (35). However, a Δ*topIB* mutant is not more sensitive to ionizing radiation than the wild type, suggesting that Topo IB does not play a crucial role in DNA repair.

Most bacteria possess two type II topoisomerases, DNA gyrase and Topo IV. DNA gyrase plays a major role in maintaining the global level of negative supercoiling of the chromosomes, while Topo IV is mainly a potent decatenase required for partitioning of the daughter chromosomes at the end of DNA replication (30, 40, 41). *D. radiodurans* lacks Topo IV. Thus, it is expected that the deinococcal DNA gyrase will also ensure the Topo IV-mediated functions in chromosome segregation. In *Mycobacterium tuberculosis* and *Mycobacterium smegmatis*, DNA gyrase is also the unique type II topoisomerase present in the cells, and it was shown that it exhibits a strong decatenase activity (42). In the experiments described here, we assayed the activities of the deinococcal DNA gyrase *in vitro*. We showed that it possessed both DNA negative supercoiling and DNA decatenation activities and that both activities were sensitive to nalidixic acid and novobiocin inhibitors. Moreover, the PprA protein, shown to interact with the GyrA subunit of DNA gyrase, was able to stimulate the DNA decatenation activity of DNA gyrase without affecting its DNA negative supercoiling activity *in vitro*. *In vivo*, the absence of PprA increased the cellular sensitivity to nalidixic acid, a GyrA inhibitor, and to novobiocin, a GyrB inhibitor, and aggravated the morphological defects observed in cells treated with novobiocin.

Furthermore, we demonstrated that novobiocin treatment leads to activation of the radiation desiccation response (RDR) in *D. radiodurans* by showing the downregulation of the DdrO repressor and the consequent upregulation of DdrB and PprA, two genes belonging to the RDR regulon. Treatment of *E. coli* and *B. subtilis* with GyrB inhibitors resulted in induction of the SOS system (38, 43) and loss of chromosomal DNA supercoiling, associated with an inhibition of gyrase activity (38, 44). A reduction of chromosome supercoiling parallels the rapid decline in DNA synthesis. Inhibition of DNA gyrase activities leads to drastic topological constraints that probably result in replication fork collapse, and DNA degradation at the stopped fork might generate an SOS signal (43). The signal that initiates RDR induction in *D. radiodurans* is not currently known. The DdrO repressor is subject to proteolytic degradation by an activated form of the metalloprotease IrrE (18, 19). The *D. radiodurans* irrE gene is constitutively expressed (45), suggesting that IrrE must be activated. The addition of novobiocin to *D. radiodurans* might result in severe modifications of DNA supercoiling and perturbation of DNA replication, as observed in *E. coli* and *B. subtilis*. Our recent results also suggest that induction of the RDR regulon may be triggered by an oxidative stressed state of the cells (19). An oxidative stressed state may result from novobiocin treatment, since it was shown that nalidixic acid and other bactericidal antibiotics generate free radicals responsible for cell killing in *E. coli* (46). An understanding of the precise mode of RDR regulon induction by novobiocin awaits further studies to identify the mechanism of RDR regulation.

After exposure to gamma radiation, the absence of PprA results in major defects in DNA segregation and cell division after completion of DNA repair (21). In *E. coli*, newly duplicated origin regions segregate to opposite sides of the cell soon after initiation of replication, while segregation of the terminus region occurs very late in the cell cycle, as the daughter cells separate (47). We recently showed that *D. radiodurans* also presents prolonged colocalization of the Ter domain of chromosome 1 (48) and that the segregation delay of the terminus is enhanced after irradiation (48). This suggests that the activities of the deinococcal DNA gyrase have to be regulated to control chromosome decatenation. The GyrA and GyrB proteins are distributed largely inside the nucleoid in nonirradiated as well as in irradiated *D. radiodurans* cells (36, 49).

After irradiation, DNA is shattered into hundreds of fragments. Even after reconstitution of circular chromosomes, irradiated cells are expected to contain a large amount of relaxed DNA that might pointlessly recruit DNA gyrase. The PprA protein, by its localization through the septum after completion of DNA repair (21, 50), might facilitate, by its interaction with GyrA, relocalization of the DNA gyrase at the septum. It has been shown that PprA polymerizes along dsDNA (23). Thus, we can also propose that PprA, like the MukB condensin in *E. coli*, may remodel the DNA and generate a preferred substrate for DNA gyrase and, thus, that the PprA-GyrA interaction might increase the effective rate of DNA decatenation. In nonirradiated cells, PprA is expressed at a low basal level and is not detected by immunofluorescence microscopy. Moreover, its absence has no effect on the viability and the morphology of cells that divide normally (21). Thus, we can imagine that, like *E. coli* Topo IV, whose decatenation activity is regulated through a physical interaction of the ParC subunit with FtsK, MreB, or MukB (51 – 53), the decatenation activity of deinococcal DNA gyrase could be regulated by its interaction with key proteins involved in chromosome segregation or cell division. FtsK and SMC (structural maintenance of chromosome), a functional analog of MukB, are present in *D. radiodurans*, but unlike the rod-shaped *Deinococcus deserti* bacteria, *D. radiodurans* bacteria do not encode a homolog of the *E. coli* MreB protein. We have previously shown that the absence of SMC in *D. radiodurans* does not disturb chromosome segregation (54). Further studies will be required for a better understanding of the regulation of chromosome segregation and cell division in *D. radiodurans*.

## MATERIALS AND METHODS

### Bacterial strains, plasmids, and DNA manipulations.

The bacterial strains used are listed in Table 2. The *E. coli* strains used were DH5α as the general cloning host and Rosetta 2(DE3)pLysS (Novagen) for protein expression. *E. coli* strains were grown at 37°C in lysogeny broth (LB) or 2× YT medium (Bio101, Inc.). All *D. radiodurans* strains were derivatives of strain R1 (ATCC 13939). They were grown at 30°C in 2× TGY (1% tryptone, 0.2% dextrose, 0.6% yeast extract) or plated on 1× TGY containing 1.5% agar. When necessary, media were supplemented with the appropriate antibiotics used at the following final concentrations: 6 µg/ml kanamycin and 3.5 µg/ml chloramphenicol for *D. radiodurans* and 30 µg/ml kanamycin, 35 µg/ml chloramphenicol, and 100 µg/ml ampicillin for *E. coli*.

**TABLE 2:** Bacterial strains

Bacterial strain	Description	Source or reference
*E. coli* strains		
DH5α	*supE44* Δ*lacU*(ϕ80*lacZ*ΔM15) *hsdR17 recA1 endA1 gyrA96 thi-1 relA1*	Laboratory stock
Rosetta 2(DE3)pLysS	F*^−^ ompT hsdS*_B_(r_B_^−^ m_B_^−^) *gal dcm λ*(DE3 [*lacI lacUV5-T7 gene 1 ind1 sam-7 nin-5*])pLysSRARE (*cat*)	Novagen
*D. radiodurans* strains		
R1	Wild type, ATCC 13939	Laboratory stock
GY12830	*ddrB*::SPA::*cat*	13
GY13338	*gyrA*::SPA::*cat*	36
GY14615	*pprA*::HA::*kan*	21
GY14641	Δ*pprA*Ω*kan gyrA*::SPA::*cat*	This work
GY14661	Δ*pprA*Ω*cat*	This work
GY14697	*pprA*::HA::*kan gyrA*::SPA::*cat*	This work
GY15985	*ΔtopIBΩkan ΔpprAΩcat*	This work
GY15987	*ΔtopIBΩkan*	This work
GY16173	*ddrO*::FLAG::*cat*	19
GY16179	*pprA*::6His::*kan*	This work

Plasmids pET26b, pET30Ek/LIC, and pET21d were used to construct vectors for overexpression of *D. radiodurans* GyrA, GyrB, and PprA, respectively (Table 3). Plasmid DNA was extracted from *E. coli* using the QIAprep spin miniprep kit (Qiagen). All constructions were verified by DNA sequencing. Relaxed pHOT-1 DNA (2.6 kb) and kinetoplast DNA (kDNA) were purchased from TopoGEN.

**TABLE 3:** Plasmids used in the study

Plasmid	Description	Reference or source
pGTC101	Source of chloramphenicol cassette	67
p11086	Source of kanamycin cassette in *D. radiodurans*	Laboratory stock
pET21d	PET expression system, pT7*lac*, C-terminal 6His tag, Amp^r^	Novagen
pET26b	PET expression system, pT7*lac*, C-terminal 6His tag, Kan^r^	Novagen
pET21d	PET expression system, pT7*lac*, N-terminal 6His tag, Kan^r^	Novagen
pET21d-*pprA*	pET21d NcoI/XhoI + PCR fragment containing *pprA*	This work
pET26b-*gyrA*	pET26b NdeI/XhoI + PCR fragment containing *gyrA*	This work
pET30EK/LIC-*gyrB*	pET30Ek/LIC + PCR fragment containing *gyrB*	This work

Δ*pprA*Ω*cat*, Δ*topIBΩkan*, and *pprA*::6His::*kan* alleles were constructed by the tripartite ligation method (55). Transformation of *D. radiodurans* with PCR products or genomic DNA was performed as previously described (13). The genetic structure and the purity of the mutants were checked by PCR.

Chromosomal DNA of *D. radiodurans* was extracted as previously described (56). PCR amplification of DNA fragments using plasmid or genomic DNA as the template was performed using Phusion DNA polymerase (Thermo Scientific) or GoTaq DNA polymerase (Promega). Oligonucleotides used in this study will be provided on request.

### Gamma irradiation treatment of *D. radiodurans*.

Exponential-phase cultures grown in 2× TGY were concentrated to an *A*_650_ of 20 in 2× TGY and irradiated on ice with a ^137^Cs irradiation system (Institut Curie, Orsay or Paris, France) at 3.8 kGy (dose rate of 40.1 Gy/min).

### Coimmunoprecipitation and Western blot analysis of the samples.

*D. radiodurans* bacteria in which a bait protein had been tagged at its C-terminal end with a SPA or HA epitope were grown in 2× TGY and exposed or not to 3.8 kGy gamma radiation. Following irradiation, cultures were diluted in 2× TGY to an *A*_650_ of 0.4 and incubated at 30°C for 110 min. Cells (50 ml) (with or without irradiation) were centrifuged, and the pellets were suspended in 600 µl of lysis buffer A (50 mM Tris, pH 7.5, 100 mM NaCl, 5 mM MgCl_2_, 1 mM dithiothreitol [DTT], 0.5% Triton X-100) with 0.1 M protease inhibitor (Pefabloc; Euromedex). Cells were disrupted with a FastPrep apparatus (FP120; Bio101, Inc.), using 0.1-g amounts of glass beads (500 µm) and three 30-s pulses (speed, 4 m/s). Cell debris was removed by centrifugation at 2,000 × *g* for 10 min at 4°C. Amounts of approximately 500 µl of supernatant were incubated with 2 µg of monoclonal antibody (mouse anti-FLAG monoclonal antibodies [Sigma-Aldrich] or mouse anti-HA monoclonal antibodies [Eurogentec]) at 10°C under gentle agitation for 2 h for immune complex formation. Amounts of 20 μl of Bio-Adembeads coated with protein G (Ademtech), washed twice in 20 µl of lysis buffer A using the Ademtech magnet, were suspended in 20 µl of lysis buffer. The washed beads were added to the supernatants treated with antibodies, and the mixtures were incubated at 10°C under gentle agitation for 1 h. The coimmunoprecipitated complexes bound to the beads were then washed five times with 500 µl of lysis buffer A using the Ademtech magnet before being suspended in 35 µl of 1× Laemmli loading dye. Samples were heated at 95°C for 5 min and replaced on the magnet, and the supernatants containing the enriched proteins were kept at −20°C before shotgun proteomics analyses (see below).

To prepare coimmunoprecipitated samples for Western blot analyses, cultures were treated with 2% formaldehyde for 20 min, followed by a treatment with 0.3 M glycine for 5 min before immunoprecipitation. Two different immunoprecipitations were performed, using a *pprA*::HA *gyrA::*SPA strain expressing the two proteins tagged with different epitopes; the cell extracts were incubated with anti-HA antibodies or with anti-FLAG antibodies and the bound proteins were separated by SDS-PAGE and immunoblotted with anti-FLAG antibodies or anti-HA antibodies, respectively. For this purpose, proteins were transferred from the gel onto a polyvinylidene difluoride (PVDF) membrane. The membrane was blocked with Tris-buffered saline (TBS) containing 0.05% Tween 20 before being incubated overnight at 4°C with a 1:5,000 dilution of mouse anti-HA monoclonal antibodies (Eurogentec) or rabbit anti-FLAG monoclonal antibodies (Sigma-Aldrich) in TBS containing 3% powdered milk, 0.05% Tween 20. After extensive washes in TBS with 0.05% Tween 20, the membrane was incubated with alkaline phosphatase-conjugated anti-mouse or anti-rabbit IgG as the secondary antibody, and bound antibodies were revealed by a colorimetric reaction. Gels were analyzed by measuring intensity profiles for each lane using Image Lab software (Bio-Rad).

### Tandem mass spectrometry and proteomic data interpretation.

The protein samples obtained by coimmunoprecipitation with the PprA::HA protein were heated at 95°C for 5 min and then loaded onto a 10% NuPAGE gel (Invitrogen) for a short electrophoresis in MOPS (morpholinepropanesulfonic acid) buffer. The proteins were briefly stained with Coomassie blue safe stain (Invitrogen). Polyacrylamide bands containing the whole enriched subproteomes were processed as previously described for further destaining and iodoacetamide treatments (57). Samples were subjected for 4 h to proteolysis at 37°C with 10 ng/µl of sequencing-grade trypsin (Roche) in 50 mM NH_4_HCO_3_ and 0.01% ProteaseMAX surfactant (Promega) to maximize peptide recovery as described previously (58). The reactions were stopped with 0.5% (final) trifluoroacetic acid. The resulting peptides (10 µl of the 40 µl generated with the procedure) were analyzed with an LTQ-Orbitrap XL hybrid mass spectrometer (Thermo Fisher) coupled to an UltiMate 3000 NanoLC system (Dionex-LC Packings) operating a reverse-phase Acclaim PepMap100 C_18_ μ-precolumn (5 µm, 100 Å, 300-µm inner diameter by 5 mm; Dionex) and a nanoscale Acclaim PepMap100 C_18_ capillary column (3 µm, 100 Å, 75 µm-inner diameter by 15 cm; Dionex) as described previously (59). Peptide mixtures (10 µl) were desalted on-line and then resolved at a flow rate of 0.3 µl per min using a 90-min gradient from 5 to 60% solvent B (0.1% HCOOH–80% CH_3_CN) with 0.1% HCOOH–100% H_2_O as solvent A. The linear trap quadrupole (LTQ)-Orbitrap XL mass spectrometer was recalibrated internally in real time with polydimethylcyclosiloxane ions generated from ambient air in the electrospray process {monoprotonated [(CH_3_)^2^SiO]^6^ with *m*/*z* of 445.120024} and operated in data-dependent mode using the TOP3 strategy as described previously (60). In brief, a scan cycle was initiated with a full scan of high mass accuracy from *m*/*z* 300 to 1,800 in the Orbitrap analyzer at a resolution of 30,000, followed by MS-MS scans in the LTQ linear ion trap on the three most abundant precursor ions, with dynamic exclusion of previously selected ions. Peak lists were generated using the Mascot Daemon software (Matrix Science), and MS-MS spectra were assigned with the MASCOT search engine (version 2.2.04; Matrix Science) as described previously (58). The in-house *D. radiodurans* protein sequence database (36) comprised 3,311 polypeptide sequences, totaling 1,006,757 amino acids. Peptides were identified with a *P* value threshold below 0.05. Protein spectral counts were normalized as described in Liu et al. (61) by systematically adding one spectral count to all experimental values. These normalized values were then compared with the Tfold or the ACfold modules of the PatternLab software (62).

### *In vivo* assay of *D. radiodurans* sensitivity to DNA gyrase inhibitors.

Cultures of exponentially growing cells at an *A*_650_ of 0.3 were serially diluted, and aliquots (10 µl) of each dilution were spotted on TGY agar supplemented or not with increasing concentrations of novobiocin or nalidixic acid. Plates were incubated at 30°C for 5 days.

### Fluorescence microscopy.

An overnight culture was diluted to an *A*_650_ of 0.07 in fresh 2× TGY medium with or without novobiocin (40 ng/ml) and incubated at 30°C with agitation (150 rpm). At different times, aliquots were removed and treated as previously described (29). DNA and membranes were stained with DAPI (4[prime],6-diamidino-2-phenylindole) (2 µg/ml) and FM 4-64 (*N*-[3-triethylammoniumpropyl]-4-{6-[4-(diethylamino) phenyl] hexatrienyl} pyridinium dibromide) (10 µg/ml), respectively. The stained cells were observed using a Leica DM RXA microscope, and images were analyzed using ImageJ software.

### Western blot analysis of proteins belonging to the RDR regulon after novobiocin treatment.

Novobiocin (40 ng/ml) was added or not to an exponential culture (*A*_650_ of 0.3) in 2× TGY medium. Cells were cultivated at 30°C, and 20-ml aliquots were centrifuged at the times indicated in Fig. 4. The pellets were suspended in 150 µl of 1× SSC (1× SSC is 0.15 M NaCl plus 0.015 M sodium citrate) buffer, and the cells were disrupted as described previously (54). After centrifugation, the protein concentration was measured (Bio-Rad protein assay dye reagent), and 5 or 10 µg of proteins was subjected to electrophoresis onto acrylamide gel. For detection of PprA::HA, DdrB::SPA, and DdrO::FLAG proteins, the tagged PprA and DdrB proteins were separated onto a 12% Tris glycine SDS-PAGE gel, and the tagged DdrO proteins were separated onto a 16% Tris Tricine SDS-PAGE gel. The proteins were then transferred onto a PVDF membrane, and the membranes were treated with anti-HA or anti-FLAG antibodies as described above.

### Purification of the PprA::6His protein.

*E. coli* Rosetta(DE3)pLysS was transformed with pET21d-*pprA* and grown in 2× YT medium (Bio101, Inc.) supplemented with ampicillin (100 µg/ml). When the cell culture reached an *A*_600_ of 1, PprA production was induced with 0.5 mM IPTG (isopropyl-β-d-thiogalactopyranoside; Sigma) for 4 h at 37°C. Cells were harvested by centrifugation, suspended in 40 ml of buffer A (200 mM NaCl, 20 mM Tris-HCl, pH 7.5), and stored overnight at −20°C. Cell lysis was completed by sonication (probe-tip sonicator; Branson). The His-tagged PprA protein was purified on a Ni-nitrilotriacetic acid (NTA) column (Qiagen, Inc.), eluted with 200 mM imidazole in buffer A, and loaded onto a Superdex 200 column (Amersham Pharmacia Biotech) equilibrated against the same buffer. The PprA protein was concentrated using Vivaspin centrifugal concentrators with a nominal molecular weight limit cutoff of 5,000 (Vivascience), flash frozen in liquid nitrogen, and stored at −80°C.

### Purification of the GyrA::6His and 6His::GyrB proteins.

Usually, to characterize the *in vitro* biochemical activities of bacterial gyrases, the GyrA and GyrB subunits are expressed and purified separately from pET vectors (63, 64). The A subunit is expressed as a C-terminal 6His protein and the B subunit as an N-terminal 6His protein. After purification of GyrA and GyrB proteins, the DNA gyrase activity is reconstituted by mixing the two subunits.

To purify the deinococcal DNA gyrase, the *gyrA* (*DR_1913*) and *gyrB* (*DR_0906*) genes were amplified from genomic DNA and ligated with DNA of vectors pET26b and pET30Ek/LIC, respectively. The resulting plasmids were introduced into *E. coli* Rosetta 2(DE3)pLysS. Transformed cells were grown in 100 ml of LB medium supplemented with 30 µg/ml kanamycin and 35 µg/ml chloramphenicol until an *A*_650_ of 0.4 to 0.6 was reached. The expression of the tagged proteins was induced by IPTG at a final concentration of 1 mM. Growth was continued overnight at 20°C. Cells were harvested by centrifugation, and the pellets were suspended in binding buffer (20 mM Tris-HCl, pH 7.8, 800 mM NaCl, 5% glycerol, 20 mM imidazole) (0.1 ml for 1 OD [optical density]) containing 0.03% Triton X-100. For the purification of 6His::GyrB, an EDTA-free protease inhibitor cocktail (Sigma) was added to the solution. The cells were disrupted using an ultrasonic cell disrupter. The disrupted suspensions were centrifuged at 4°C (13,000 × *g* for 20 min), and the supernatants were loaded onto 0.5-ml Ni-NTA columns (Qiagen) equilibrated with binding buffer. The columns were washed initially with 5 ml of binding buffer, followed by 4 elution steps with 5 ml of a solution containing 20 mM Tris-HCl, pH 7.8, 200 mM NaCl, and 5% glycerol and increasing concentrations of imidazole (40 mM, 60 mM, 100 mM, and 200 mM). The purity of the proteins was verified by SDS-gel electrophoresis, and the protein fractions were pooled and dialyzed on a PD10 column against 3.5 ml of 50 mM Tris-HCl, pH 7.8, 200 mM NaCl, and 30% glycerol according to the manufacturer’s protocol (GE Healthcare). The protein solutions were transferred to fresh precooled tubes, and DTT and EDTA were added at a final concentration of 1 mM. The GyrA::6His and 6His::GyrB proteins were aliquoted and stored at −80°C.

### DNA supercoiling and decatenation assays.

DNA supercoiling activity was assayed with the recombinant *D. radiodurans* GyrA and GyrB proteins and relaxed pHOT DNA (TopoGEN) as the substrate. Supercoiled pHOT is a derivative of plasmid pBR322. The relaxed form is prepared by the manufacturer using high-purity Topo I relaxation reactions, followed by inactivation and repurification of the relaxed product. The reaction mixture (20 µl) contained 35 mM Tris-HCl (pH 7.8), 24 mM KCl, 4 mM MgCl_2_, 2 mM DTT, 1 mM ATP, 1.8 mM spermidine, 100 µg/ml bovine serum albumin, 6.5% glycerol, relaxed pHOT DNA (125 ng), and GyrA and GyrB proteins in equal molar amounts. To test the effect of PprA, increasing amounts of PprA (or the equivalent volumes of buffer) were added to the reaction mixtures. The mixture was incubated for 1 h at 37°C, and the reaction was terminated by the addition of 2 µl 10× BBSE buffer (5% SDS, 100 mM EDTA, 50% glycerol, and 0.4% bromophenol blue). The samples were loaded onto a 1.2% agarose gel in TEP buffer (36 mM Tris-HCl, pH 7.8, 30 mM NaH_2_PO_4_, 1 mM EDTA) and run for 3 h at 50 V. The gel was stained with ethidium bromide (1 µg/ml) for 30 min. The bands were then visualized and quantified using Image Lab (Bio-Rad) software.

For the decatenation assay, the reaction mixture was the same as those used in the supercoiling assay, except that the DNA substrate was replaced with kinetoplast DNA (kDNA; a network of ≈5,000 catenated DNA minicircles and ≈25 maxicircles that is isolated from trypanosomatid parasites [65]) (125 ng; TopoGEN), and the incubation was performed at 37°C for 3 h. The products were then analyzed as described for the DNA supercoiling assay.

The same *in vitro* DNA negative supercoiling and DNA decatenation assays were performed in the presence of increasing concentrations of novobiocin or nalidixic acid.
